# Novel citation-based search method for scientific literature: application to meta-analyses

**DOI:** 10.1186/s12874-015-0077-z

**Published:** 2015-10-13

**Authors:** A. Cecile J W Janssens, M. Gwinn

**Affiliations:** Department of Epidemiology, Rollins School of Public Health, Emory University, 1518 Clifton Road NE, Atlanta, Georgia 30322 USA; Department of Clinical Genetics/EMGO Institute for Health and Care Research, Section Community Genetics, VU University Medical Center, Amsterdam, The Netherlands

**Keywords:** Citation, Co-citation, Literature search, Meta-analysis, Systematic review, Keywords

## Abstract

**Background:**

Finding eligible studies for meta-analysis and systematic reviews relies on keyword-based searching as the gold standard, despite its inefficiency. Searching based on direct citations is not sufficiently comprehensive. We propose a novel strategy that ranks articles on their degree of co-citation with one or more “known” articles before reviewing their eligibility.

**Method:**

In two independent studies, we aimed to reproduce the results of literature searches for sets of published meta-analyses (*n* = 10 and *n* = 42). For each meta-analysis, we extracted co-citations for the randomly selected ‘known’ articles from the Web of Science database, counted their frequencies and screened all articles with a score above a selection threshold. In the second study, we extended the method by retrieving direct citations for all selected articles.

**Results:**

In the first study, we retrieved 82 % of the studies included in the meta-analyses while screening only 11 % as many articles as were screened for the original publications. Articles that we missed were published in non-English languages, published before 1975, published very recently, or available only as conference abstracts. In the second study, we retrieved 79 % of included studies while screening half the original number of articles.

**Conclusions:**

Citation searching appears to be an efficient and reasonably accurate method for finding articles similar to one or more articles of interest for meta-analysis and reviews.

**Electronic supplementary material:**

The online version of this article (doi:10.1186/s12874-015-0077-z) contains supplementary material, which is available to authorized users.

## Background

Meta-analysis is an increasingly popular statistical method for comparing and summarizing the results of multiple independent studies. First introduced to clinical research in the 1980s, meta-analysis is now a cornerstone of evidence-based medicine [1]. It has also become an important step in establishing the credibility of research findings, such as those from hypothesis-free discovery research studies [2]. The number of published meta-analyses indexed in PubMed is increasing by about 20 % per year (PubMed).

An ideal meta-analysis provides a complete representation of all relevant data, both published and unpublished. Finding eligible studies is often the most challenging and time-consuming phase in conducting a meta-analysis, especially when the terminology for key concepts, variables and outcomes differs among studies. The Cochrane Collaboration— internationally regarded for its rigorous approach to meta-analyses of clinical interventions—recommends searching multiple publication databases by using Boolean combinations of all possible keywords, including synonyms and related words that authors may have used to describe their studies, and complementing keyword-based searches with hand screening of references listed in the retrieved articles [3]. Casting a wide net often retrieves thousands of publications that must be screened to find a handful of eligible studies. Despite its inefficiency, this approach remains the gold standard.

Finding eligible studies by screening the references and subsequent citations of articles that are already known could be seen as a way to crowd-source expert knowledge of the published scientific literature. The network properties of scientific citations have been studied extensively since the 1950s, when they were used to create the Science Citation Index [4, 5]; they have been further exploited in the development of online research tools such as *Web of Science*, *Scopus* and *Google Scholar*. Some current research explores the use of computational algorithms to automate citation retrieval for systematic reviews [6].

Although it is intuitively appealing, backward and forward citation checking falls short as a way to identify eligible articles for meta-analysis. Searching these ‘direct’ citations could be an efficient strategy only if eligible studies consistently cited all relevant earlier work, thus creating a single citation network, but this is often not the case. For example, a review of 259 meta-analyses found that in fewer than half (46 %) were included articles connected in a single citation network; in the remainder, included articles were in either two (39 %) or three or more (15 %) disconnected citation networks [7]. Citation searching has thus gained only equivocal support, even as a complement to keyword searching [8, 9].

Searching based on direct citations is insensitive and inefficient because researchers tend to cite only some related earlier articles, not all. Although eligible studies may be only sparsely connected by direct citations, taking indirect connections into account can help identify additional studies. For example, two eligible studies that are not connected by direct citations might both be co-cited by the same newer article [10], or they may be coupled because they both cite the same earlier article [11]. These citing and cited articles may be commentaries, reviews or original research articles on related topics.

The principles of co-citation and bibliographic coupling are used extensively in bibliometrics and scientometrics to document and visualize similarity between articles, topics, authors and disciplines [12–15]; however, they have not been used specifically to find eligible studies for meta-analyses or systematic reviews. We propose a search method that ranks articles on their degree of co-citation with one or more known articles and demonstrate that other studies eligible for inclusion in the meta-analysis rank high on this list.

## Methods

### The method

**Fig. 1:**
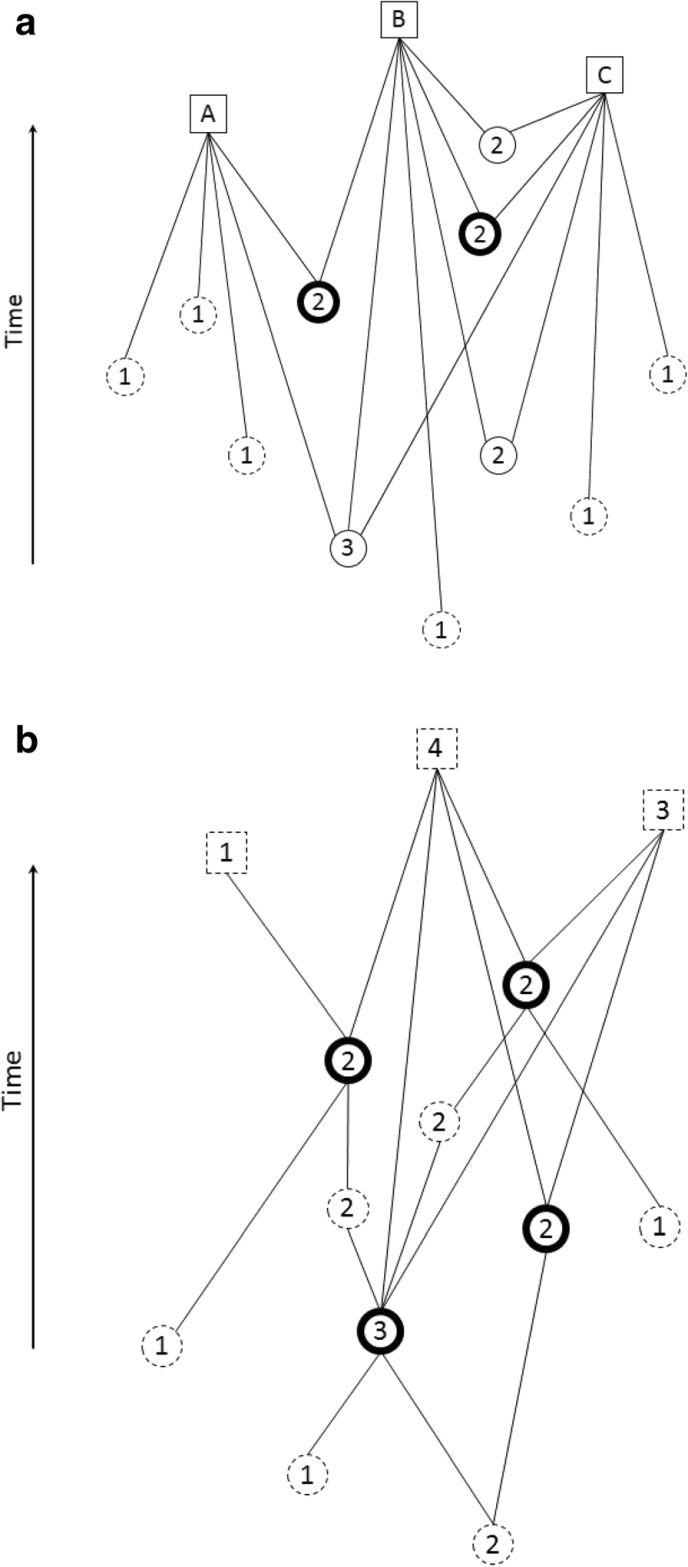
Overview of the search method. **a** Indirect citations (co-citations). Bold circles represent articles known at the beginning of the search. Squares represent citing articles; the articles on their reference lists (co-citing articles) are represented by circles. Numbers within circles indicate the number of times an article is co-cited (dashed circles represent articles co-cited only once). **b**. Direct citations. Bold circles represent articles known at the beginning of the search. Dashed squares represent citing articles; dashed circles represent articles on the known articles’ reference lists. Numbers within dashed squares and circles indicate the number of times an article cites or is cited by a known article

The search method assumes that one or more eligible studies are “known” at the start of the search (Fig. 1a, bold circles). In the event that researchers are unfamiliar with the topic, they can first perform a keyword-based search to find one or more studies that meet the inclusion criteria. When a known study is cited (Fig. 1a, squares), the reference list of the citing article contains articles co-cited with the known study (Fig. 1a, regular circles). If a known study is cited 50 times, for example, there will be 50 such reference lists. For each article on a reference list, we can count how frequently it appears on the other 49 lists. The higher the number, the more often the article was co-cited with the known study. Likewise, when two known articles are cited 50 times each, there are up to 100 reference lists. Articles that appear most frequently on these lists are the ones that were co-cited most often with one or both of the known articles. We hypothesized that limiting the screening of articles to those that were frequently cited together with one or more known articles might be an efficient method for finding other eligible studies.

We investigated the method by using Web of Science to reproduce the set of studies included in two independently selected samples of recently published meta-analyses. First we conducted a pilot study (Study 1) that applied the method to ten meta-analyses. We investigated the performance of the method by comparing different selection thresholds and examined the types of studies that were not retrieved. In the second study (Study 2), we used results from the first study to fine-tune the selection threshold (see below) and augmented the search strategy with a second search based on direct citations, specifically to retrieve recent articles that had not been cited yet.

### Study 1

#### Selection of meta-analyses

Meta-analyses were identified by two different PubMed searches: Eight meta-analyses by searching on a single title word (“meta-analysis”) and two by searching a specific journal name (“Cochrane Database Syst Rev”). Meta-analyses were selected consecutively and were eligible if they had reported the total number of articles that were retrieved by applying one or more search strategies to one or more databases. This number, which indicated the total number of articles that had been screened for eligibility in the meta-analysis, could be reported in a flowchart or in the text, but should have been reported separately from the number of full-text articles screened (we noticed that this distinction was ambiguous in many meta-analyses). All procedures and analyses described below were performed separately for each of the ten meta-analyses. A short description of the meta-analyses is provided in Additional file 1: Table S1.

#### Selection of “known” articles

From each meta-analysis, we randomly chose one or two included studies to start the search. After drawing citation networks (Additional file 1: Figure S1), we discovered that for two meta-analyses, we had chosen a study that would favor our results: the study of O’Keefe et al. in the meta-analysis of Frolkis et al. [16], which was part of a second citation network, and the study of Gallon et al. in the meta-analysis of Knoll et al. [17], which was not connected to any other study. We decided not to consider these studies “known” but to investigate whether they would be retrieved by searching from the one remaining study.

#### Obtaining citation networks

To illustrate the density of the citation networks, we obtained all direct citations between the studies included in the meta-analyses. Using Web of Science (Thomson Reuters, USA), we manually screened the reference lists of all published studies included in the meta-analysis and documented for each article which of the other included studies were cited. Citation networks were drawn manually (Additional file 1: Figure S1).

#### Data collection

The known articles (Fig. 1a, bold circles) were identified in the Web of Science database. Articles that cited a known article (which Web of Science calls “citing articles”; Fig. 1a, squares A, B, C) were saved to the “Marked list”. This list was downloaded with the full bibliographic details of each article, including the cited references (regular and dashed circles), and saved in a Microsoft Excel file. The list of citing articles naturally includes the published meta-analysis. We removed the meta-analysis itself and all articles with a more recent publication date from the list and excluded them from the rest of the analyses.

Web of Science provides the entire reference list for each citing article in a single cell. To obtain a full list of all co-cited articles, we extracted the references from all citing articles into a single datasheet. Any article that is cited by multiple citing articles appears more than once on the datasheet; the number of times it appears is its co-citation frequency or co-citation strength. The co-citation frequency has a minimum value of 1 and a maximum value equal to the number of citing articles. We counted and collapsed duplicate records, sorted the articles in descending order of co-citation frequency, and marked all articles that were included in the original meta-analysis.

#### Analyses

We quantified the performance of the search method using three different selection strategies to screen the co-citations: (1) those that were co-cited at least once (threshold ≥1, which was the entire dataset; Fig. 1a, regular circles); (2) those that were co-cited at least twice (threshold ≥2); and (3) those that were frequently co-cited with the known articles (varying the threshold among meta-analyses). We decided to examine frequently co-cited articles after exploring the distributions of co-citations; we learned that for each meta-analysis, about 80 % of the articles are co-cited once and only a limited number are co-cited frequently (Additional file 1: Figure S2). We chose a threshold for each meta-analysis such that the number of frequently co-cited articles was between 100 and 150, or closest to 100 when the nearest thresholds were both outside that range. The chosen threshold varied among meta-analyses, depending on the citation density (for highly-cited topics, the threshold could be set higher).

For each published meta-analysis, as a measure of the efficiency of the method, we counted the number of articles selected at each threshold and compared this with the number of articles screened by the authors of the meta-analysis. As a measure of the accuracy of the method, we also counted the number of studies that had been included in the meta-analysis and compared this with the total number of articles included in the meta-analysis at each selection threshold.

### Study 2

#### Selection of meta-analyses

We searched PubMed using the title word “meta-analysis” to identify meta-analyses published between 1 January and 28 February 2015 in journals that were listed in the category of Core Clinical Journals. This search yielded 121 articles. We sorted the list on journal name and selected the first meta-analysis for each journal, which yielded 49 meta-analyses. Seven meta-analyses were excluded either because they had not performed a literature search (*n* = 4; e.g., genome-wide association studies), provided only one flowchart for multiple meta-analyses (*n* = 2), or reported a search for more recent articles to update a previously published meta-analysis (*n* = 1). A short description of the meta-analyses is provided in Additional file 1: Table S1.

#### Selection of “known” articles

For each meta-analysis, we used a standardized procedure to select two included studies. We surmised that researchers who consider performing a meta-analysis know of at least two studies and are more likely to be familiar with the studies that had larger sample sizes. We therefore assumed for this analysis that the two largest studies indexed in Web of Science were known and that literature searches were performed to find all the others. When the largest studies were not indexed (e.g., because they were published in journals that were not indexed, in theses or on websites; *n* = 11), we took the next largest. Choosing the largest study might seem to bias the results in our favor; however, the largest studies were often not the first, and were therefore not published in high-impact journals or were too recent to have been cited. Both of these conditions would tend to undermine the observed accracy of our method. On the other hand, when the largest studies were highly cited, choosing them would tend to reduce the method’s observed efficiency.

#### Data collection and analyses

The literature search in Study 2 consisted of two searches: first for co-citations and second for direct citations. The first search was identical to the procedure in Study 1, except that we applied a different selection threshold to improve efficiency in the case of highly-cited articles in dense citation networks. In this case, in addition to the simple count of the number of times an article was co-cited with the known articles, we calculated an index (the *j-index*) that represented the number of times the article was co-cited as percentage of the number of citing articles. We then selected for screening all articles that were co-cited more than once and co-cited in more than 1 % of the citing articles. Thus, the screening threshold was based on the number of citing articles: when the number of citing articles was less than 100, the threshold was based on the number of co-citations; when it was more than 100, it was based on the index.

For the second search, we extracted all backward and forward direct citations (Fig. 1b, dashed squares and circles, regular circles) for the two known articles and all articles that were retrieved in the first search (Fig. 1b, bold circles). We counted the frequency of each citation in the database and ranked the citations in descending order. All articles that had two or more direct citations were screened to find the articles that were included in the meta-analysis but not retrieved in the first search.

## Results

### Study 1

The meta-analyses included between 4 and 27 studies (median 10) for which the authors had screened from 784 to 17,500 articles (median 1,642; Table 1). The number of direct citations connecting the included studies ranged from 2 to 99 (median 15; Additional file 1: Figure S1) with a median of 2 citations between any two articles (data not shown). In three meta-analyses, all included studies were connected in a single citation network; the other meta-analyses included between one and seven disconnected studies, i.e., articles that did not cite and were not cited by any of the other articles in the direct citation network (Additional file 1: Figure S1). Among the 10 meta-analyses, the number of articles co-cited with the known articles ranged from 588 to 8,388 (median 997; Table 1), producing a much richer network of indirect connections than the sparse network of direct connections.

**Table 1:** Articles screened and retrieved in the replication of ten published meta-analyses

	Original meta-analysis		All co-citations				All co-cited >1				Frequently co-cited			
First author	Articles screened	Studies included	Articles screened		Studies retrieved		Articles screened		Studies retrieved		Articles screened		Studies retrieved	
Boothe [27]	17,500	8	5,595	(32)	8	(100)	913	(5)	8	(100)	109	(1)	8	(100)
Frolkis [16]	9,151	12	967	(11)	10	(83)	224	(2)	7	(58)	108	(1)	6	(50)
Oliver-Williams [28]	8,646	10	588	(7)	8	(80)	62	(1)	5	(50)	62	(1)	5	(50)
Knoll [17]	2,365	21	7,638	(323)	19	(90)	1,719	(73)	18	(86)	132	(6)	11	(52)
Stevanovic [29]	2,090	13	987	(47)	12	(92)	186	(9)	10	(77)	77	(4)	10	(77)
De Vries [30]	1,194	9	8,388	(703)	9	(100)	1,924	(161)	9	(100)	124	(10)	8	(89)
Crider [31]	1,154	5	1,006	(87)	5	(100)	120	(10)	5	(100)	120	(10)	5	(100)
Herretes [32]	898	4	670	(75)	3	(75)	111	(12)	3	(75)	111	(12)	3	(75)
Gharaibeh [33]	836	27	880	(105)	26	(96)	173	(21)	21	(78)	116	(14)	19	(70)
Gu [34]	784	6	3,234	(413)	6	(100)	780	(99)	6	(100)	129	(16)	5	(83)
Median	1,642	10	997	(81)	9	(94)	205	(11)	8	(82)	110	(8)	7	(76)

Percentages are shown in parentheses; values greater than 100 indicate that more articles were selected for screening than in the original meta-analysis. “Frequently co-cited” refers to citations above a threshold in the ranked list that was chosen such that 100–150 articles needed to be screened (See Methods; Additional file 1: Figure S2)

We evaluated three different selection criteria for screening co-citations. Screening all co-citations retrieved 75 to 100 % (median 94 %) of all studies included in the original meta-analyses (Table 1). This selection was more efficient than the original search, except when the known articles were highly cited (cited > 100 times). Screening only the articles that were co-cited more than once with known articles was more efficient than the original search for 9 of 10 meta-analyses (Table 1), retrieving a median of 82 % of included studies while screening a median of 11 % as many articles. Screening only the frequently co-cited articles (see definition in Methods and Additional file 1: Figure S2) reduced the number of screened articles to between 1 and 16 % (median 8 %) of the original number and retrieved 50 to 100 % (median 76 %) of the included studies.

We reviewed the titles of articles that ranked highest in co-citation frequency for each meta-analysis and found that they tended to refer to the same topic (see examples in Additional file 1: Table S2); also the articles that were not included or cited in the meta-analysis. Topics were more diverse among articles that were co-cited fewer times. This is most apparent in Additional file 1: Table S2D, where the titles of articles that were co-cited two or three times had little in common with the topic of the meta-analysis.

The types of articles that were not found by our method varied, as expected, according to the selection criteria. (Table 2). Most of the articles that were not co-cited or co-cited only once were either published in non-English languages, published before 1975, published very recently, or available only as abstracts.

**Table 2:** Characteristics of studies included in published meta-analyses that were not retrieved by citation-based literature search at each selection threshold

	All co-citations	All co-cited >1	Frequently co-cited
Retrieved	106	92	80
Missed	9 (5)	14 (6)	12 (7)
Abstract	2 (0)	0 (0)	1 (0)
Non-English language	1 (0)	6 (1)	0 (0)
Old publication (<1975)	2 (2)	2 (0)	1 (0)
Recent publication (2014)	2 (2)	1 (1)	0 (0)
Other	2 (1)	5 (5)	10 (7)
Total	115	106	92

Legend: The ten meta-analyses included 115 studies, of which 106 were retrieved by our search. Of those, 92 were co-cited more than once and 80 appeared in the list of frequently co-cited articles. The headings of the table refer to the thresholds presented in Table 1. The numbers in parentheses indicate how many articles had direct connections with other articles in the meta-analysis, because they were either citing or cited by those articles. These numbers indicate whether the articles could have been found by adding a search for direct citations, as was done in Study 2. For example, five of the nine studies that were missed in the first selection were citing or cited by other articles included in the meta-analysis

Co-citation searching identified 49 of 55 articles that were not connected with the known articles via direct citations (Table 3), including 15 of 19 articles that were completely disconnected from the entire single citation network surrounding the known articles (Additional file 1: Figure S1).

**Table 3:** Retrieval of articles that had no direct connections to the known articles

Published meta-analysis	Number of articles without direct connections	Retrieved in:		
		All co-citations	All co-cited > 1	Frequently co-cited
Boothe [27]	1	1	1	1
Frolkis [16]	8	7	5	4
Oliver-Williams [28]	5	3	0	0
Knoll [17]	16	14	14	7
Stevanovic [29]	4	4	2	2
De Vries [30]	4	4	4	2
Crider [31]	1	1	1	1
Herretes [32]	0	0	0	0
Gharaibeh [33]	14	13	8	6
Gu [34]	2	2	2	1
Total	55	49	37	24

The table summarizes data presented in Additional file 1: Figure S1. For example, in the meta-analysis of Boothe et al. [27], only one article included in the meta-analysis had no direct connection with either of the two known studies. That article was frequently co-cited and was thus identified at any of the three thresholds

### Study 2

**Fig. 2:**
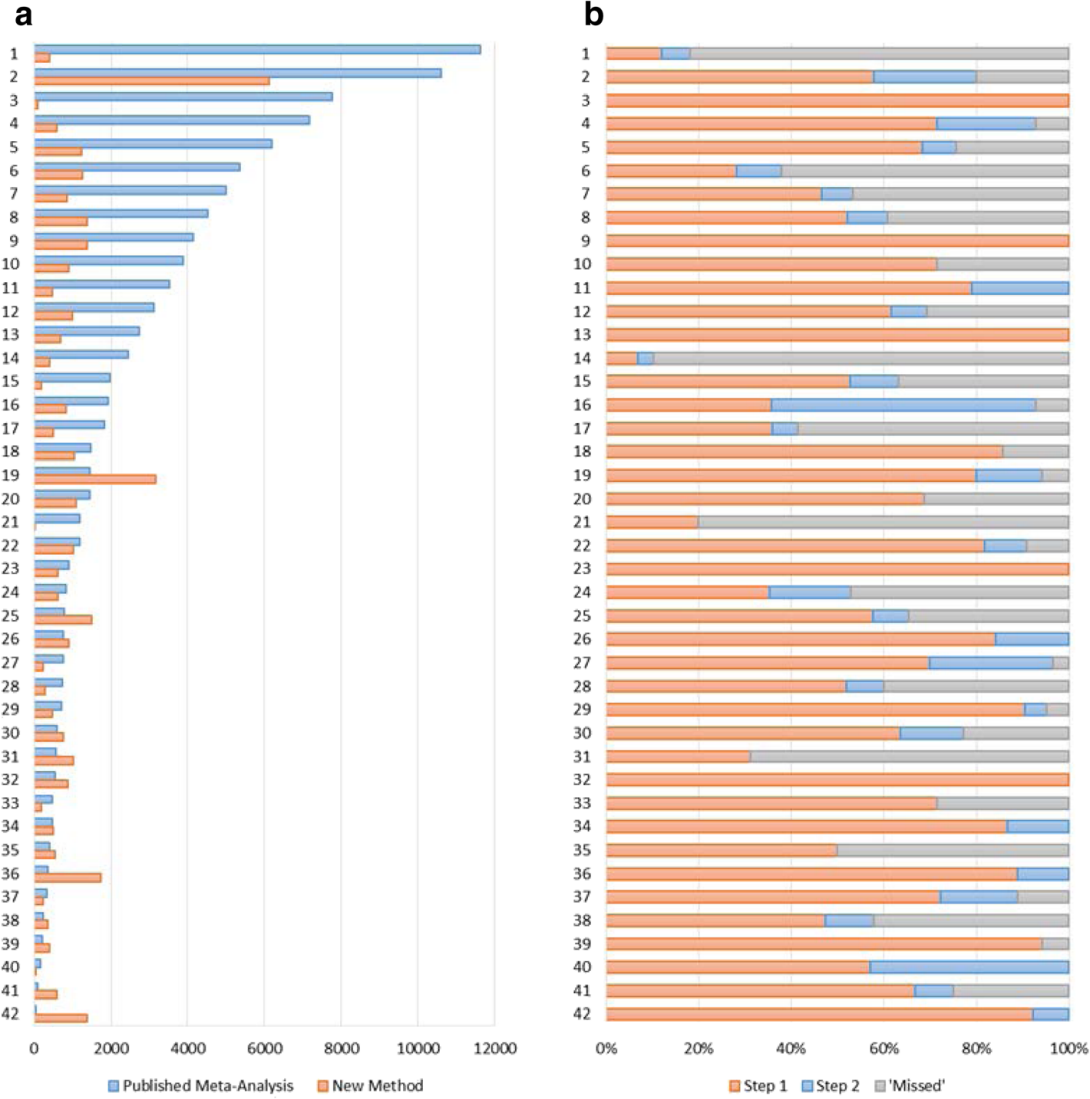
Articles screened and studies retrieved in Study 2. **a**. Number of articles screened for the published meta-analysis, compared with the number selected for screening by the new method (searches for indirect and direct citations combined). **b** Studies retrieved in Study 2 (searches for indirect and direct citations combined) as percent of the number of studies included in the published meta-analysis (numbered as in Fig. 2a)

**Fig. 3:**
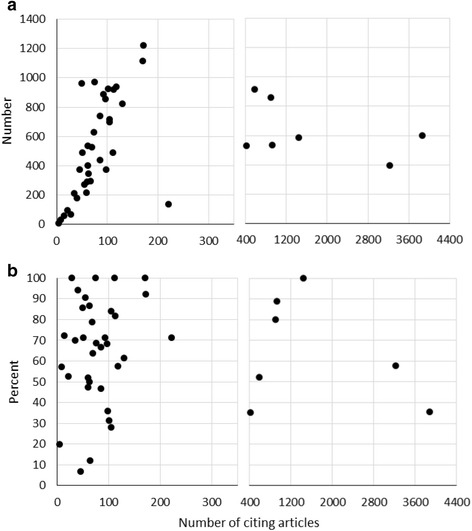
Articles screened and studies retrieved in Study 2 (indirect citations), in relation to the number of citing articles. **a** Number of articles screened. **b** Studies retrieved (percent)

We conducted a second study of 42 different meta-analyses, in which we applied a standardized strategy consisting of two consecutive searches. The first search was the same as in the Study 1, except that we screened all articles that were co-cited in more than 1 % of the citing articles. In the first search, we retrieved a median of 69 % of the included articles while screening only 29 % of the number of articles that the authors of the meta-analyses had screened (Table 4; Fig. 2). A higher number of citing articles increased the number of articles that needed to be screened (Fig. 3a) without markedly increasing the number of studies retrieved (Fig. 3b).

**Table 4:** Number of articles screened and retrieved in Study 2

	Original meta-analysis			Indirect citations (search 1)				Indirect and direct citations (search 1 + 2)			
	Articles screened	Studies included	Citing articles	Articles screened		Studies retrieved		Articles screened		Studies retrieved	
Mehrabi [35]	4,148	29	170	1,113	(27)	29	(100)	1,383	(33)	29	(100)
Pathak [36]	543	6	1,437	584	(108)	6	(100)	886	(163)	6	(100)
Viswanathan [37]	2,749	6	74	627	(23)	6	(100)	689	(25)	6	(100)
Vrablik [38]	7,771	3	28	68	(1)	3	(100)	81	(1)	3	(100)
vanWely [39]	894	18	106	444	(50)	18	(100)	615	(69)	18	(100)
Schuit [40]	39	13	171	1,221	(3,131)	12	(92)	1,385	(3,551)	13	(100)
Deng [41]	362	9	928	533	(147)	8	(89)	1,726	(477)	9	(100)
Nwachuku [42]	464	15	62	404	(87)	13	(87)	502	(108)	15	(100)
Gu [43]	764	19	104	719	(94)	16	(84)	908	(119)	19	(100)
SanLorenzo [44]	3,529	19	67	296	(08)	15	(79)	468	(13)	19	(100)
Al-Wassia [45]	166	7	8	32	(19)	4	(57)	52	(31)	7	(100)
Elshaer [46]	750	30	35	210	(28)	21	(70)	235	(31)	29	(97)
Mumme [47]	701	21	55	271	(39)	19	(90)	468	(67)	20	(95)
Hazlewood [48]	1,463	35	897	861	(59)	28	(80)	3,162	(216)	33	(94)
Sheyin [49]	221	17	40	180	(81)	16	(94)	392	(177)	16	(94)
Yuan [50]	7,175	14	51	490	(7)	10	(71)	596	(8)	13	(93)
Elmariah [51]	1,934	14	3,870	599	(31)	5	(36)	836	(43)	13	(93)
Cheelo [52]	1,192	11	112	919	(77)	9	(82)	1,017	(85)	10	(91)
Gu [53]	326	18	14	59	(18)	13	(72)	233	(71)	16	(89)
Saleh [54]	1,480	14	49	964	(65)	12	(86)	1,055	(71)	12	(86)
Emdin [55]	10,598	45	3,223	395	(4)	26	(58)	6,116	(58)	36	(80)
Sayegh [56]	594	22	69	529	(89)	14	(64)	759	(128)	17	(77)
Kamper [57]	6,189	41	96	857	(14)	28	(68)	1,227	(20)	31	(76)
Taioli [58]	98	24	85	441	(450)	16	(67)	595	(607)	18	(75)
Sharpe [59]	3,875	7	92	886	(23)	5	(71)	911	(24)	5	(71)
Zhang [60]	468	7	221	140	(30)	5	(71)	198	(42)	5	(71)
Siddiqui [61]	3,119	13	129	824	(26)	8	(62)	1,002	(32)	9	(69)
Mair-Jenkins [24]	1,449	32	75	971	(67)	22	(69)	1,086	(75)	22	(69)
Bonitsis [62]	795	52	117	937	(118)	30	(58)	1,489	(187)	34	(65)
Williams [23]	1,976	19	21	95	(5)	10	(53)	186	(9)	12	(63)
Souto [63]	4,527	23	580	913	(20)	12	(52)	1,372	(30)	14	(61)
Zhen [64]	742	25	59	215	(29)	13	(52)	290	(39)	15	(60)
Shan [65]	243	19	60	289	(119)	9	(47)	344	(142)	11	(58)
Marcuzzi [66]	5,009	15	85	739	(15)	7	(47)	851	(17)	8	(53)
Lipinski [67]	824	17	420	531	(64)	6	(35)	610	(74)	9	(53)
Stevens [68]	400	6	62	536	(134)	3	(50)	551	(138)	3	(50)
Bernstein [69]	1,837	53	98	376	(20)	19	(36)	505	(27)	22	(42)
Avni [20]	5,365	103	104	698	(13)	29	(28)	1,259	(23)	39	(38)
Kumar [21]	573	16	101	926	(162)	5	(31)	1,013	(177)	5	(31)
Fazeli [22]	1,195	5	4	7	(1)	1	(20)	7	(1)	1	(20)
Brydges [19]	11,628	33	63	347	(03)	4	(12)	391	(3)	6	(18)
McNally [18]	2,453	88	45	374	(15)	6	(7)	399	(16)	9	(10)
Median	1,194	18	85	530	(29)	12	(69)	652	(50)	13	(79)
Mean	2,396	23	336	539	(58)^a^	13	(65)	901	(90)^a^	15	(75)

Values in parentheses are the number of articles screened or studies retrieved as percentages of the numbers in the original meta-analyses. ^a^Calculated after removing outlier [40]

In the second search, we obtained the direct citations of all articles retrieved in the first search and screened those that cited or were cited by two or more of them. The second search retrieved an additional 10 % of the included studies, which brought the median to 79 % (Table 4; Fig. 2). The two searches combined required screening 50 % as many articles as had been screened by authors of the original meta-analyses.

## Discussion

Before discussing the implications of our method, several methodological issues about the studies needs to be discussed. First, we evaluated the performance of our method conservatively by assuming that the original meta-analyses were comprehensive and complete. Thus, when we failed to retrieve a study, we considered it a shortcoming of our method, not of the published meta-analysis. Yet, in the meta-analysis of second surgery in Crohn’s disease, for example, we missed the only two pediatric studies [16], and we missed five articles that were published before 1975 (Table 2); these studies may be less comparable to others included in the meta-analysis. Furthermore, for all meta-analyses, we found original articles on the same topic that were more frequently co-cited than the articles that were included (see examples in Additional file 1: Table S2); however, we did not attempt to investigate whether they had been excluded after screening or perhaps should have been included in the meta-analyses.

Second, our method demonstrated lower efficiency and accuracy in the second study, which could be attributed to several factors. The second study included more highly cited topics, which tend to generate a higher number of co-citations, thus reducing efficiency. This study also included more meta-analyses for which the authors screened a relatively low number of articles. In the first study, none of the meta-analyses had screened fewer than 500 articles and only three (30 %) had screened fewer than 1,000 (Table 1); in contrast, of the 42 meta-analyses in the second study, 10 (24 %) had screened fewer than 500 articles and 20 (48 %) had screened fewer than 1,000 (Table 4).

The second study also included more meta-analyses on heterogeneous topics, which tended to reduce accuracy. For example, we retrieved only 10 % of the studies included in a meta-analysis on normalization of vitamin D levels in children of various ages and with various diseases [18]; 18 % of the studies on the use of simulation-based assessments for patient-related outcomes for a variety of tasks and skills in physicians, medical students, dentists and nurses [19]; and 38 % of the studies on the safety of intravenous iron preparations in patients with various disorders [20]. Clearly, the method does not work when the topic of the meta-analysis is heterogeneous and the studies of interest are unlikely to have cited each other. The second study also included several meta-analyses with very small sample sizes, including one in which half of the studies were case reports that had few or no references [21], as well as a meta-analysis for which the ‘known’ studies were cited only four times in total [22]. The percentage of retrieved studies jumped to 89 % when these five meta-analyses were excluded.

And third, we compared our method with literature searches of the published meta-analyses that often combined separate searches in multiple databases, supplemented with the screening of references lists, conference abstracts and grey literature, and the consultation of experts. These additional strategies may have yielded studies that were not indexed in databases like Web of Science or Medline, and contributed to underestimation of the accuracy. For example, we were unable to retrieve the two master theses that were included in a meta-analysis for which the authors searched the Dissertation Abstracts International database, [23] and missed many South-American and Asian studies of a meta-analysis for which the authors additionally searched the LILACS and KOREAMED databases [20]. Additional strategies like these can be used to complement our search method--either to find more eligible studies or to increase confidence in the results of the search method when no other studies are found.

Using a citation-based search to identify articles for meta-analysis has several advantages. Perhaps most importantly, the quality of the search does not depend on keywords, which is particularly relevant for topics where there is no consistent terminology. In contrast to machine-learning algorithms, citation-based searching does not depend on the quality and selection of a training set. Co-citation searching was more efficient than keyword-based searching, retrieving a median of 76 % of eligible studies from a short list of around 100 of the most frequently co-cited articles (Table 1). Co-citation searching also retrieved articles published in journals that were not indexed in Web of Science, suggesting that the need to search other databases could be reduced. An interesting example is the meta-analysis of immunoglobulin treatment for severe acute respiratory infections such as SARS, avian influenza (H1N1), and the Spanish influenza of 1918 [24]. This meta-analysis included 16 studies published in 1919–1920, of which we were able to retrieve 13. These included publications in the Norsk Magazin för Laegevidenskapen, Boston Medical and Surgical Journal, La Presse Médicale, New York Medical Journal and Hygiea, which are all journals that no longer exist. These studies could be retrieved because they had been cited by studies of more recent outbreaks that were published in journals that were indexed in Web of Science.

The accuracy and efficiency of co-citation searching depends on characteristics of the underlying citation network. By design, our method misses the studies that the collective community of researchers apparently did not find worth citing. In our analysis, these included abstracts, articles in non-English languages, very old articles, and publications in semi-scientific journals, reports, websites, and theses. In addition, some newer and some very old articles were not cited often enough to rank high in our search. Some modifications of our method could help identify these articles; for example, as shown in Table 2, half of the missed articles were connected with retrieved articles through direct citations. Aggregating and ranking the direct citations among all articles that are retrieved by our search might be an efficient way to find them. Other modifications might be necessary when the method is applied to topics with very dense citation networks of highly-cited articles; in these situations the number of articles to be screened could be limited further, for example, by setting a higher citation threshold.

## Conclusions

Reviewing published scientific findings requires evaluating unstructured data and text, for which human insight and judgment are crucial [25, 26]. Our method makes use of the collective knowledge of researchers in a given field by performing an initial ranking that can be fully automated. Researchers conducting meta-analyses must still identify and evaluate the eligible studies, but with the advantage of being able to screen only half of the number of articles compared to keyword-base literature search, and to screen the most similar articles first. Although we evaluated this method as it applies to meta-analysis, it could be used to find related articles for any type of study, as demonstrated in Additional file 1: Table S2. Screening a short list of frequently co-cited articles is an efficient strategy for finding key articles related to one or more “known” articles, even when a formal meta-analysis is not the goal. Going forward, this strategy has the potential to help strengthen connections among articles and improve and facilitate the process of evidence synthesis.

## Additional file

Additional file 1: (DOCX 975 kb)
