# Erratum to: Novel citation-based search method for scientific literature: application to meta-analyses

**DOI:** 10.1186/s12874-015-0093-z

**Published:** 2015-11-10

**Authors:** A. Cecile J. W. Janssens, M. Gwinn

**Affiliations:** Department of Epidemiology, Rollins School of Public Health, Emory University, 1518 Clifton Road NE, Atlanta, Georgia 30322 USA; Department of Clinical Genetics/EMGO Institute for Health and Care Research, Section Community Genetics, VU University Medical Center, Amsterdam, The Netherlands

## Erratum

Unfortunately, the original version of this article [1] contained an error.

Under ‘Study 2’, the text read: “The first search was the same as in the Study 1, except that we screened all articles that were co-cited in more than 1 % of the citing articles.”

However, this sentence should have also included articles that were “co-cited more than once”, to read: “The first search was the same as in the Study 1, except that we screened all articles that were co-cited more than once and co-cited in more than 1 % of the citing articles.”

We apologise for this error.

## References

[1] Janssens ACJW, Gwinn M. BMC Med. Res. Methodol. 2015;15:84. doi:10.1186/s12874-015-0077-z. 10.1186/s12874-015-0077-z PMC4604708 26462491

