# Supplementary material for: Novel citation-based search method for scientific literature: application to meta-analyses
Source: BMC Med Res Methodol. 2015 Oct 13;15:84. doi: 10.1186/s12874-015-0077-z (PMC4604708; doi:10.1186/s12874-015-0077-z)

**Additional file 1**

**Novel Citation-based Search Method for Scientific Literature:**

**Application to Meta-Analyses**

A. Cecile J.W. Janssens*, M. Gwinn

*Corresponding author. E-mail: cecile.janssens@emory.edu

This file includes:

Tables S1 and S2

Figs. S1 and S2

**Table S1.** Characteristics of included meta-analyses in Study 1 and 2.

Legend: The meta-analyses are grouped by study and ranked in descending order of observed accuracy. Topics were extracted from the title or abstract. Type of study characterizes the main research question: ‘intervention’ refers to studies that compared the efficacy and safety of drug therapies, behavioral therapies, medical procedures and services, ‘association’ to studies that investigated the relationship between two variables, ‘incidence’ and ‘prevalence’ for assessment of disease frequencies, and ‘descriptive’ for general assessment of health outcomes such as quality of life and function. Study selection was inferred from the methods section of the articles. A distinction was made between randomized controlled trials (RCT), randomized and nonrandomized trials (labeled as ‘trials’), observational studies, which included case-control and cohort studies, and case reports.

|  | **Topic** | **Type** | **Study selection** |
| --- | --- | --- | --- |
| **Study 1** |  |  |  |
| Boothe [27] | Residential traffic exposure and childhood leukemia | Association | Observational |
| De Vries [30] | Statin and secondary prevention of cardiovascular and cerebrovascular events | Intervention | RCT |
| Crider [31] | Prenatal folic acid and asthma | Association | RCT/Observational |
| Gu [34] | Oscillatory ventilation in acute respiratory distress syndrome | Intervention | RCT |
| Knoll [17] | Sirolimus and malignancy and survival after kidneytransplantation | Intervention | RCT |
| Gharaibeh [33] | Medical interventions in traumatic hyphema | Intervention | RCT |
| Stevanovic [29] | Desflurane and outcomes after laryngeal mask airway anesthesia | Intervention | RCT |
| Herretes [32] | Corticosteroids in bacterial keratitis | Intervention | RCT |
| Frolkis [16] | Cumulative incidence of second intestinal resection in Crohn’s disease | Incidence | Observational |
| Oliver-Williams [28] | Miscarriage and future maternal cardiovascular disease | Association | Observational |
| **Study 2** |  |  |  |
| Mehrabi [35] | Laparoscopic versus open distal pancreatectomy for lesions in the pancreas | Intervention | Trials and observational |
| Pathak [36] | Risk of bleeding with apixaban in patients with renal impairment | Intervention | RCT |
| Viswanathan [37] | Medication therapy management interventions in outpatient settings | Intervention | Trials and observational |
| Vrablik [38] | Beside ocular ultrasonography and retinal detachment | Diagnosis | Observational |
| vanWely [39] | Ultrasound-guided biopsy in patients with extensive axillary tumor burden in breast cancer | Diagnosis | Observational |
| Schuit [40] | Progestogens and perinatal outcome in twin pregnancies | Intervention | RCT |
| Deng [41] | Defibrillator or resynchronization therapy in patients with heart failure | Intervention | Trials |
| Nwachuku[42] | Somatosensory evoked potential changes during carotid endarterectomy | Diagnosis | Trials and observational |
| Gu[43] | Lung-protective ventilation and clinical outcomes among patients undergoing surgery | Intervention | RCT |
| SanLorenzo[44] | Melanoma in airline pilots and cabin crew | Incidence | Observational |
| Al-Wassia[45] | Umbilical cord milking at birth | Intervention | RCT |
| Elshaer[46] | Subtotal cholecystectomy for gallbladders | Intervention | Trials and observational |
| Mumme[47] | Medium-chain triglycerides and weight loss and body composition | Intervention | RCT |
| Hazlewood[48] | Immunosuppressants and biologics and remission in Crohn’s disease | Intervention | RCT |
| Sheyin[49] | Troponin elevation in patients with sepsis | Prognosis | Observational |
| Yuan[50] | Intracranial pressure monitoring and mortality in patients with traumatic brain injury | Intervention | RCT and observational |
| Elmariah[51] | Dual antiplatelet therapy and mortality | Intervention | RCT |
| Cheelo[52] | Paracetamol in pregnancy and asthma in child | Intervention | Observational |
| Gu[53] | Etomidate and mortality in patients with sepsis | Intervention | RCT and observational |
| Saleh[54] | Glycopeptides versus β-lactams and surgical site infections | Intervention | RCT |
| Emdin[55] | Blood pressure lowering in type 2 diabetes | Intervention | RCT |
| Sayegh[56] | Nonsurgical treatment and longitudinal outcomes of lateral epicondylitis | Intervention | RCT |
| Kamper[57] | Biopsychosocial rehabilitation in chronic low back pain | Intervention | Trials |
| Taioli[58] | Pleurectomy decortication versus extrapleural pneumonectomy in mesothelioma | Intervention | Observational |
| Sharpe[59] | Indoor fungal diversity and asthma | Association | Observational |
| Zhang[60] | Fractional flow reserve versus angiography in percutaneous coronary intervention | Intervention | Observational |
| Siddiqui[61] | Mesh sacrocolpopexy versus native tissue vaginal repair | Intervention | RCT |
| Mair-Jenkins[24] | Convalescent plasma and hyperimmune immunoglobin for severe acute respiratory infections | Intervention | Case reports, trials and observational |
| Bonitsis[62] | Gender differences in Adamantiades-Behcet’s disease | Association | Observational |
| Williams[23] | Depression after spinal cord injury | Prevalence | Observational |
| Souto[63] | Lipid profile changes following biological agents and tofacitinib in chronic inflammatory arthritis | Intervention | RCT |
| Zhen[64] | Infrared tympanic thermometry and fever in children | Diagnosis | Observational |
| Shan[65] | Quality of life after total knee replacement | Descriptive | Observational |
| Marcuzzi[66] | Somatosensory function in spinal pain | Descriptive | Trials and observational |
| Lipinski[67] | Troponin in patients with chest pain | Diagnosis/prognosis | Observational |
| Stevens[68] | Ondansetron and postoperative tramadol | Intervention | RCT |
| Bernstein[69] | Diabetes and hypertension in homeless adults | Prevalence | Observational |
| Avni[20] | Safety of intravenous iron preparations | Intervention | RCT |
| Kumar[21] | Nasopharyngeal airway stenting devices for obstructive sleep apnea | Intervention | Case reports |
| Fazeli[22] | Biofeedback for daytime voiding disorders in children | Intervention | RCT |
| Brydges[19] | Simulation-based educational assessments and patient-related outcomes | Intervention | Trials and observational |
| McNally[18] | Rapid normalization of vitamin d levels | Treatment | Trials |

**Table S2.** Titles of the 25 most frequently co-cited articles for four meta-analyses included in the pilot study (Table 1).

Legend: Percentage of retrieved studies refers to the proportion of studies in the published meta-analysis that were retrieved when the co-citation threshold was set at >1 (Table 1). Count represents the number of times the article was co-cited with the known articles (see Supplementary Methods). Titles have been shortened to the key topic or variables that were studied. Colors indicate whether the articles was included, cited by, or not mentioned in the meta-analysis.

| **Color** | **Interpretation** |
| --- | --- |
|  | “Known” article at start of the search |
|  | Included in the meta-analysis |
|  | Cited by but not included in the meta-analysis |
|  | Not cited or included in the meta-analysis |

Table S2A. Residential traffic exposure and childhood leukemia [27]

Percentage of retrieved studies: 100%

| **Title** | **Count** |
| --- | --- |
| Exposure to benzene, other hydrocarbons and acute childhood leukemia | 62 |
| Residential exposure to traffic and childhood cancer | 53 |
| Road traffic and childhood leukemia | 44 |
| Air pollution from traffic and childhood cancer | 35 |
| Traffic density and risk of childhood leukemia | 29 |
| Exposure motor vehicle exhaust and childhood cancer | 27 |
| Traffic density in proximity to home and childhood leukemia | 25 |
| Residential traffic density and childhood cancer | 25 |
| Proximity to main roads, petrol stations and incidence of childhood cancer | 24 |
| Traffic patterns and incidence childhood cancer | 21 |
| Hazardous air pollutants and childhood cancer | 19 |
| Residence next to petrol stations/garages and acute childhood leukemia | 15 |
| Exposure to gasoline and childhood leukemia | 15 |
| Exposure to fine particulate air pollution and lung cancer | 14 |
| Air pollution and childhood cancer | 14 |
| Hazard proximities of childhood cancers | 12 |
| Residential traffic density and cancer incidence | 12 |
| Residential traffic density and childhood leukemia | 11 |
| Nitrates in drinking water and mortality risk from childhood brain tumors | 11 |
| Traffic air pollution and childhood leukemia | 11 |
| Environmental risk factors of childhood leukemia | 10 |
| Spatial epidemiology: methods and applications | 10 |
| Exposure to household solvents and childhood acute leukemia | 10 |
| Oil combustion and childhood cancers | 10 |
| Hazardous air pollutants and childhood cancer | 10 |

Table S2B. Medical interventions in traumatic hyphema (TH) [33]

Percentage of retrieved studies: 78%

| **Title** | **Count** |
| --- | --- |
| Steroids in TH | 33 |
| Topical aminocaproic acid in TH | 23 |
| Aminocaproic acid in TH | 22 |
| Corticosteroids in TH | 20 |
| Management of TH | 19 |
| Monocular versus binocular patching in TH | 18 |
| Surgical versus medical treatment in TH | 18 |
| Atropine in TH | 17 |
| Urokinase in TH | 15 |
| Epsilon-aminocaproic acid in TH | 14 |
| Clinical characteristics and estrogens in TH | 14 |
| Observations on TH | 14 |
| TH in children | 13 |
| Tranexamic acid in TH | 13 |
| Aminocaproic acid versus prednisone in TH | 12 |
| Aminocaproic acid in TH | 12 |
| TH in urban population | 12 |
| Rebleeding after TH | 12 |
| Pathogenesis and management of TH | 12 |
| Report of 200 cases with TH | 11 |
| Epsilon-aminocaproic acid in TH | 10 |
| Treatment of TH | 10 |
| Aminocaproic acid in TH | 10 |
| Rebleeding after TH | 10 |
| Management of TH in children | 10 |

Table S2C. Second intestinal resection in Crohn's disease (CD) [16]

Percentage of retrieved studies: 58%

| **Title** | **Count** |
| --- | --- |
| Outcomes of patients with CD | 34 |
| Clinical course in CD | 16 |
| Medical treatment and surgical resection rates in CD | 14 |
| Clinical course in CD | 13 |
| Surgical rates and medical management of CD | 13 |
| Incidence and surgical rates in inflammatory bowel disease | 13 |
| Hospitalizations and surgery in CD | 12 |
| Incidence and prevalence of inflammatory bowel disease | 12 |
| Disease activity courses in CD | 9 |
| Disease behavior in CD | 8 |
| Therapies and outcomes in inflammatory bowel disease | 8 |
| Cost analysis of inflammatory bowel disease | 8 |
| Natural history of CD | 8 |
| Clinical course in ulcerative colitis | 8 |
| Hospitalization, surgery and readmission in inflammatory bowel disease | 7 |
| Need for intestinal surgery in CD | 7 |
| Incidence and early disease course in inflammatory bowel disease | 7 |
| Hospitalization rates for inflammatory bowel disease | 7 |
| Recurrence rates in CD | 7 |
| Postoperative recurrence in CD | 6 |
| Hospitalization rates for inflammatory bowel disease | 6 |
| Behavior of CD | 6 |
| Hospitalization and CD | 5 |
| Azathioprine vs conventional management in CD | 5 |
| Surgical treatment and long-term prognosis in CD | 5 |

Table S2D. Miscarriage and future maternal cardiovascular disease (CVD) [28]

Percentage of retrieved studies: 50%

| **Title** | **Count** |
| --- | --- |
| Menstrual, reproductive factors and risk of myocardial infarction | 10 |
| Pregnancy loss and CVD risk | 9 |
| Pre-eclampsia and CVD risk | 4 |
| Age at menarche, parity, age at first birth and risk of coronary heart disease | 4 |
| Menstrual and reproductive risk factors for ischemic heart disease | 4 |
| Age at menarche, menopause, reproductive year and risk of CVD mortality | 4 |
| Reproductive history and CVD risk | 4 |
| Pre-eclampsia, recurrent pregnancy loss and cardiovascular events | 4 |
| Association of age at menarche with CVD risk factors | 4 |
| Number of pregnancies and CVD risk | 4 |
| Reproductive factors and risk of myocardial infarction | 4 |
| Pregnancy complications and risk of ischemic heart disease | 4 |
| Loss of early pregnancy and risk of ischemic heart diesase | 4 |
| Parity and CVD risk | 3 |
| Age at menarche, reproductive years, menopause on CVD risk factors | 3 |
| Menarcheal age and adult obesity | 3 |
| Age of menarche and metabolic syndrome | 3 |
| Age at menarche, and mortality from ischemic heart disease and stroke | 3 |
| Pre-eclampsia/eclampsia and cardiovascular sequelae | 3 |
| 30-year recall of early menstrual history | 3 |
| Age at menarche and adult BMI | 3 |
| Early menarche and development of CVD risk factors | 3 |
| Danish National Hospital Register | 2 |
| Chlamydia pneumonia infected macrophages | 2 |
| Chlamydia trachomatis; infection in pregnancy sequelae | 2 |

**Figure S1.** Citation networks for the ten meta-analyses included in Study 1.

Legend: Graphs (Fig. S1a-f) present the citation networks for the ten meta-analysis searches that were reproduced in our study. The studies included in the meta-analyses are referred to by the name of the first author and year of publication and are sorted by publication year, with the most recent publications at the top of each page. Boxes indicate studies that were assumed to be known at the start of the search. The numbers in parentheses indicate the number of times that an article was cited together with one of the studies that was assumed to be known at the start. The studies with an asterisk (*) are not connected to the known studies through direct citations (Table 3 reports whether these studies were retrieved using our method). Red arrows indicate the direction of the citations. The total number of direct citations between all studies included in the meta-analysis is indicated in right lower corner.

Figure S1a
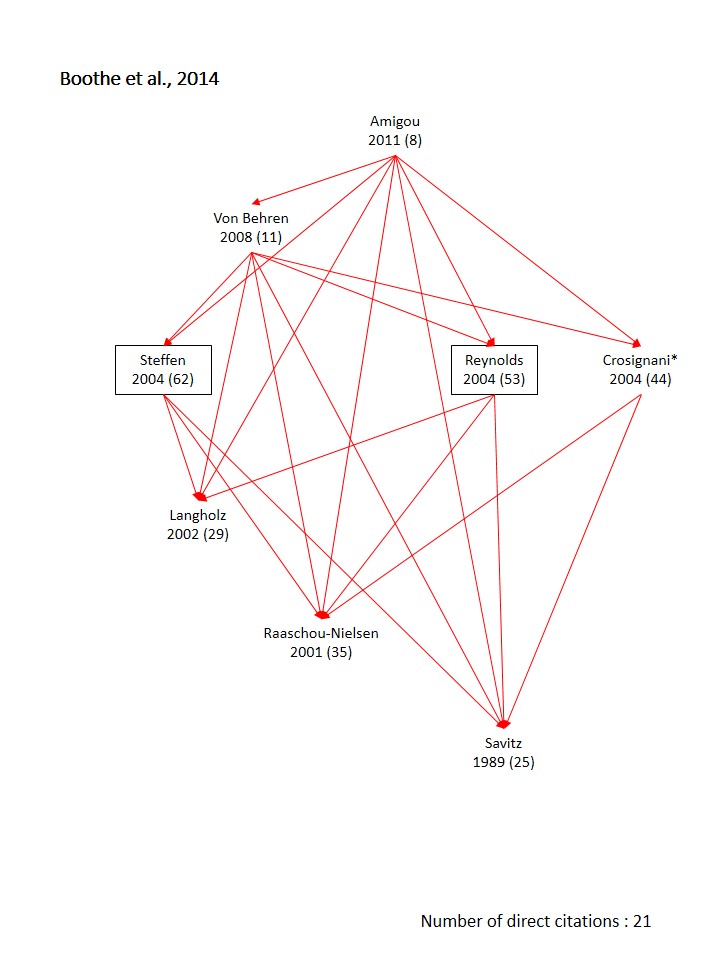


Figure S1b
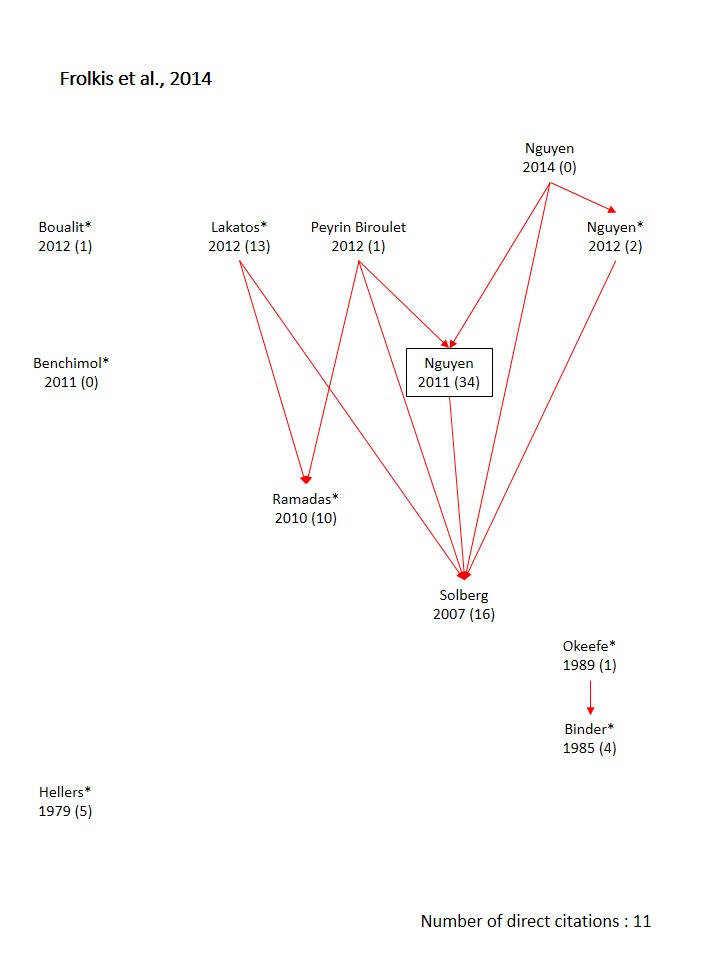


Figure S1c


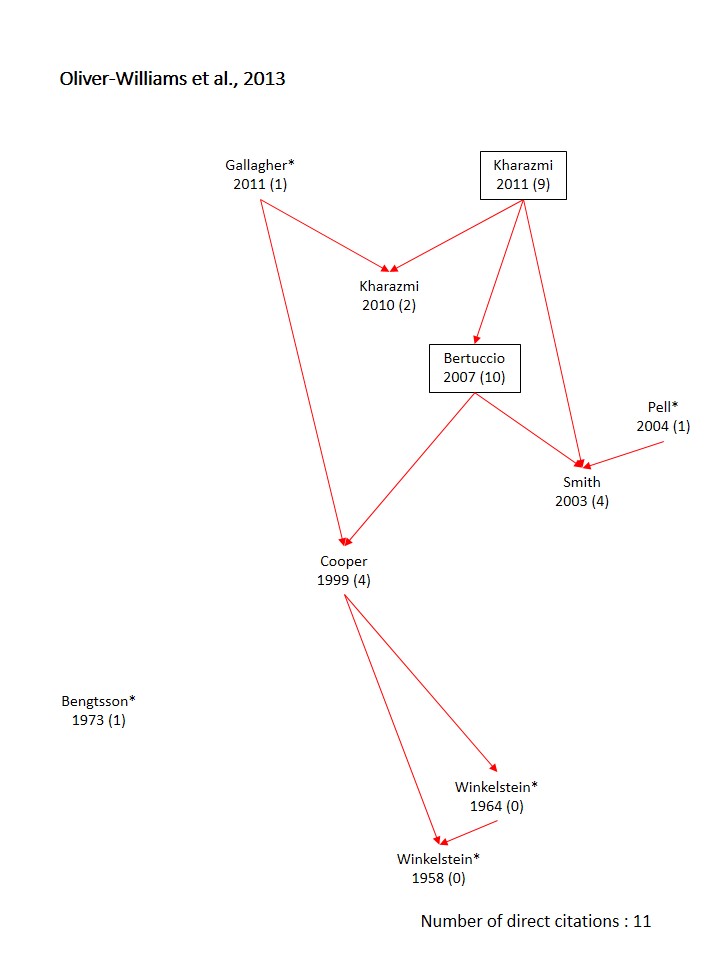


Figure S1d
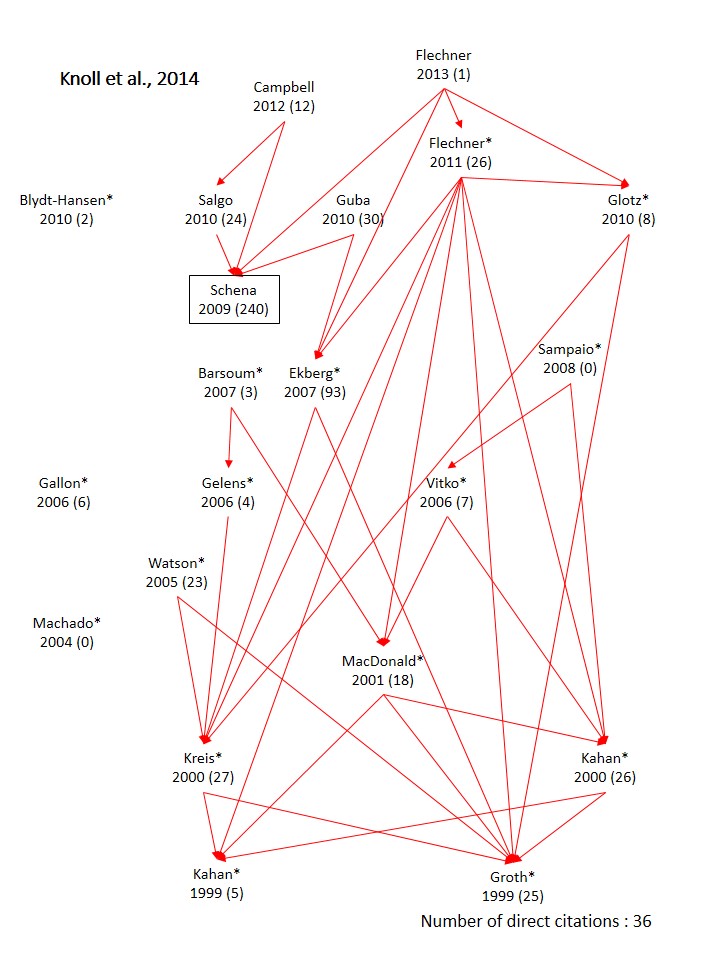


Figure S1e
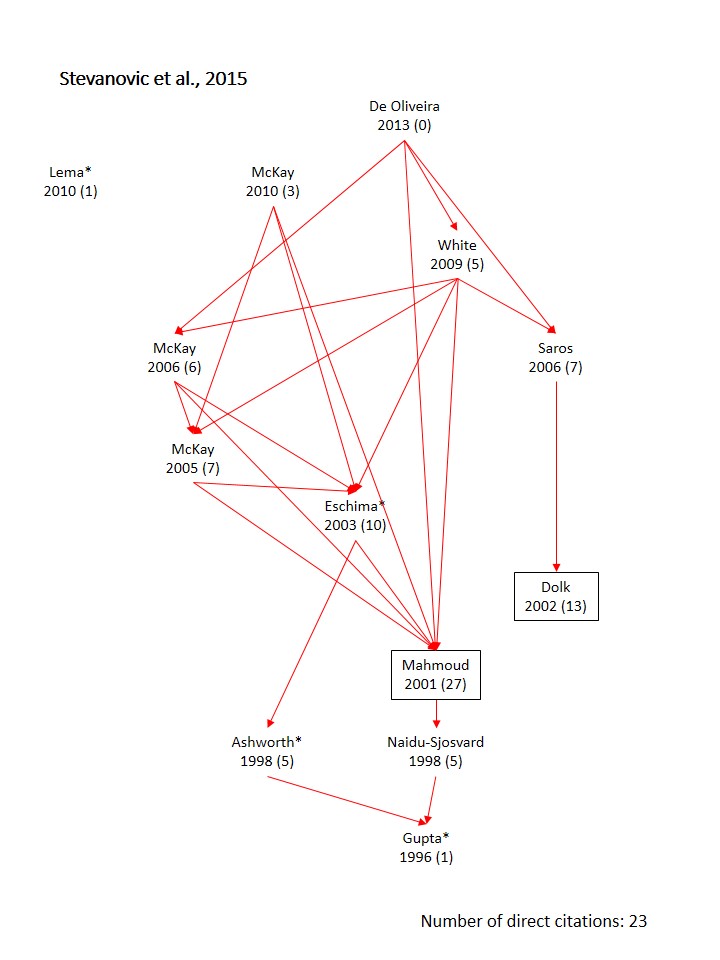


Figure S1f
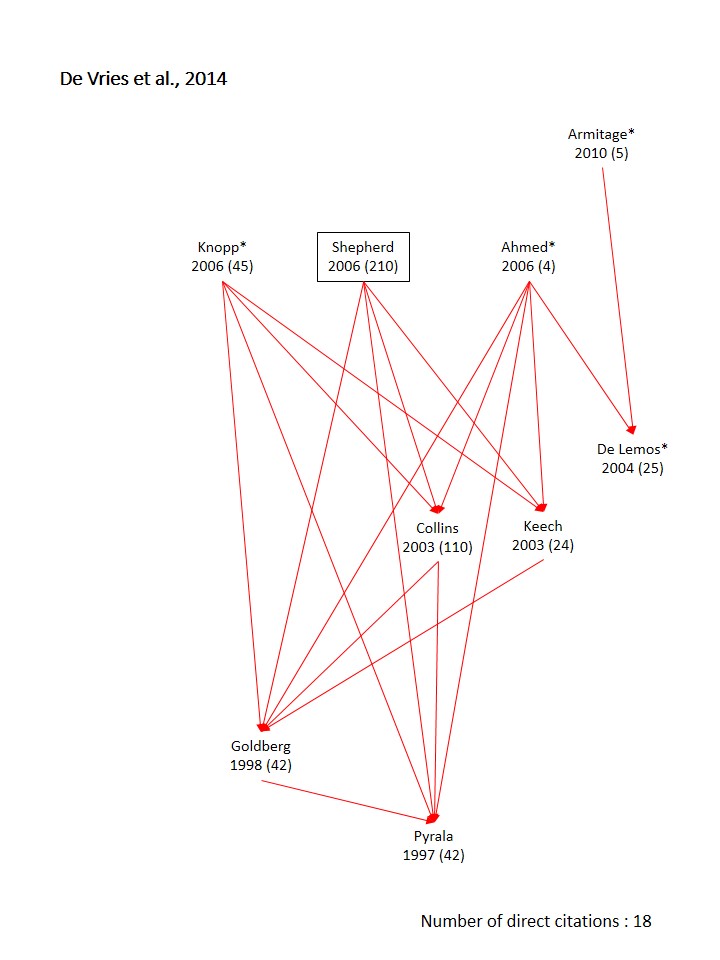


Figure S1g
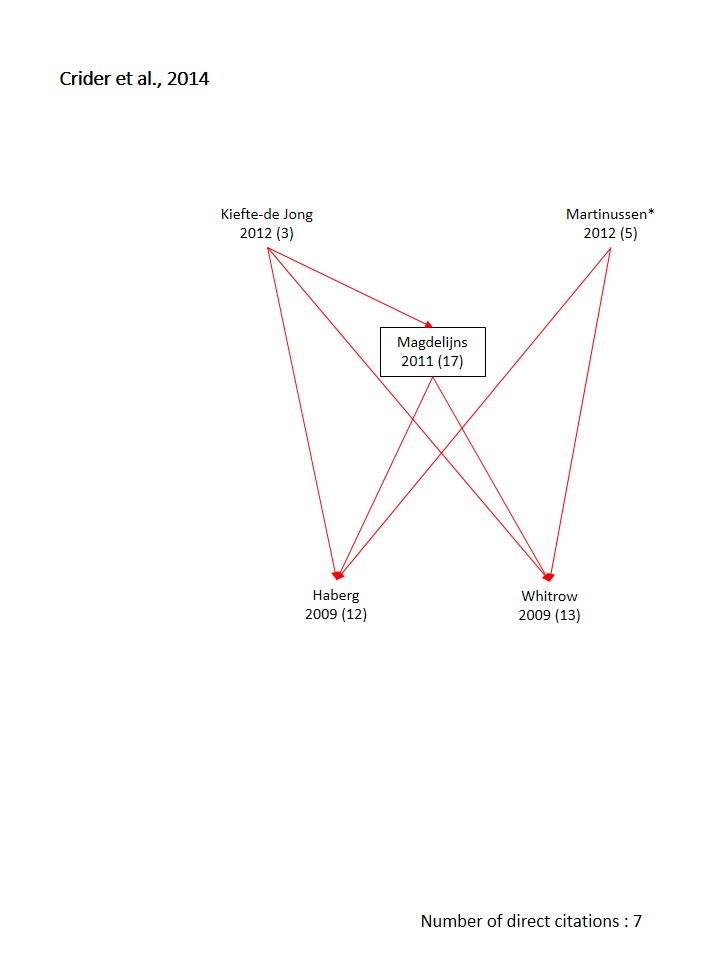


Figure S1h
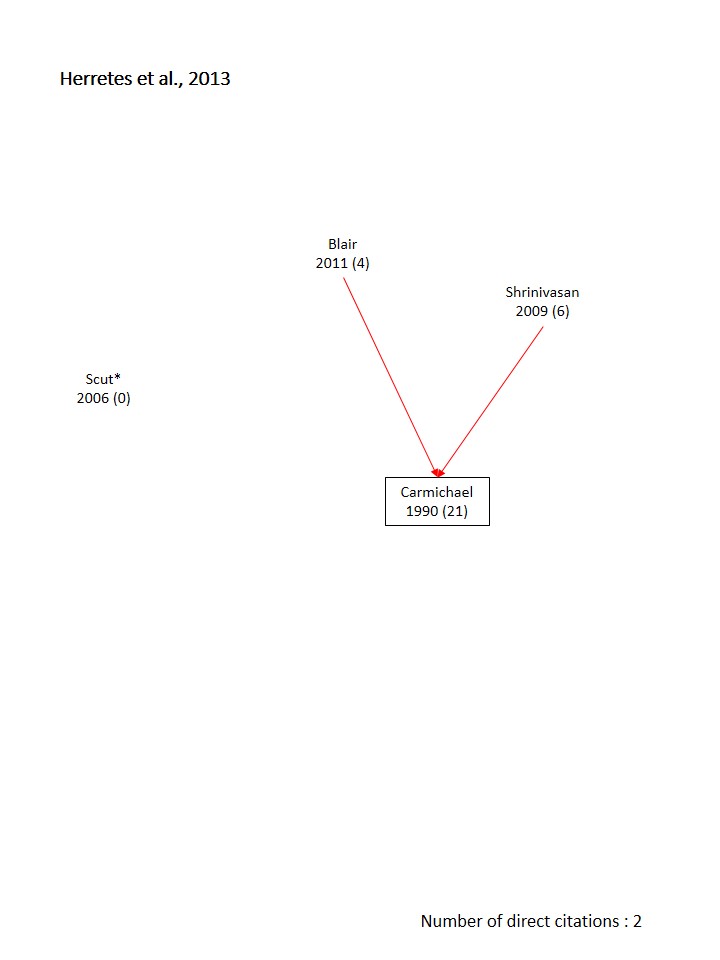


Figure S1i
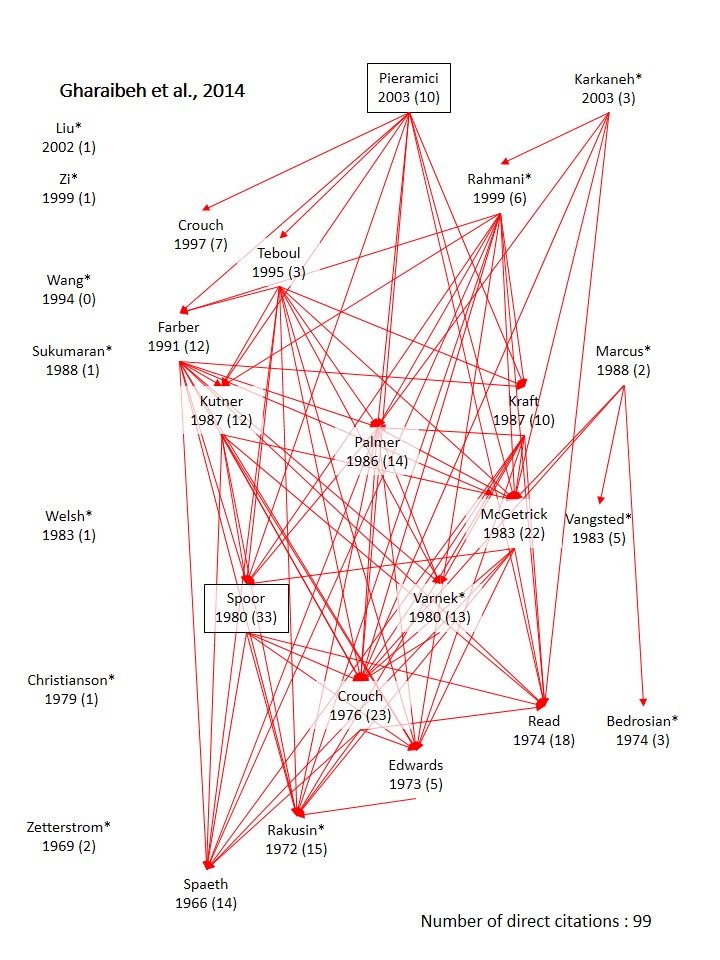
 Figure S1j
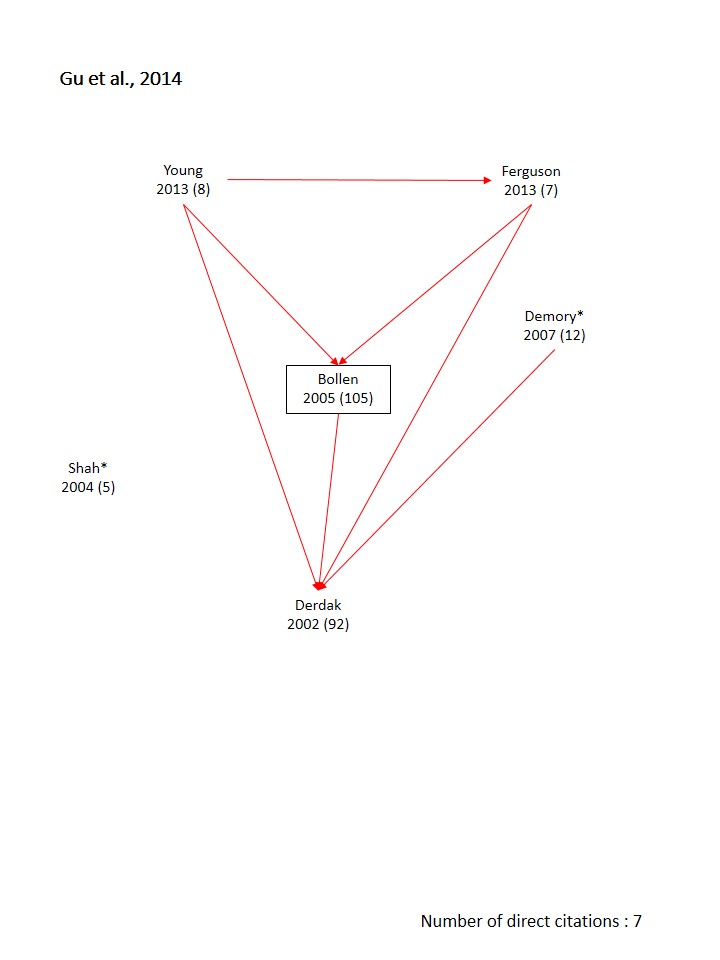


**Figure S2.** Frequency distribution of the number of co-citations for the ten meta-analyses included in Study 1.

Legend: The figure indicates how frequently each of the articles was co-cited with the known articles for the ten meta-analyses in Study 1 [16,17, 28-3]. The dashed lines indicate the thresholds at which articles were considered to be frequently co-cited with the known articles. This threshold was determined from the total number of articles (in parentheses) and their co-citation frequencies. The thresholds were chosen in such a way that approximately 100-150 articles would need to be screened.


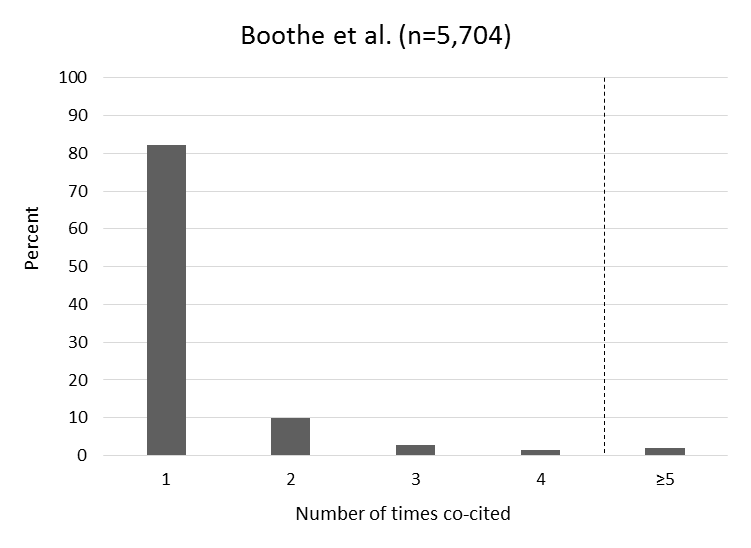

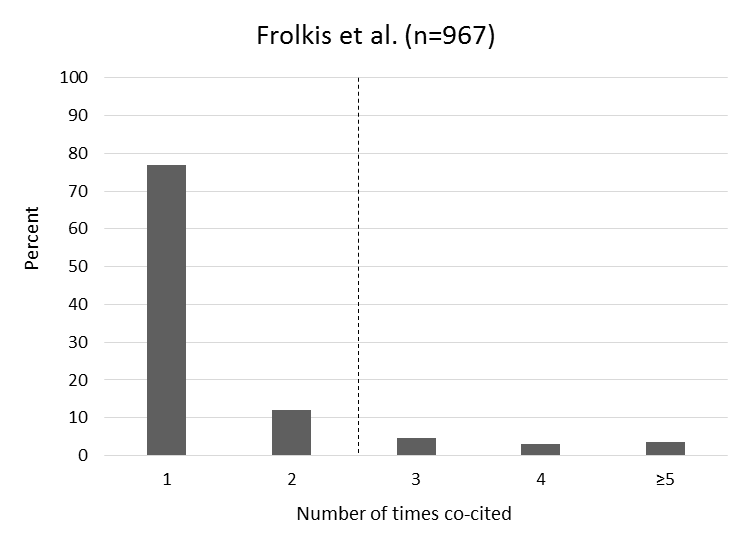

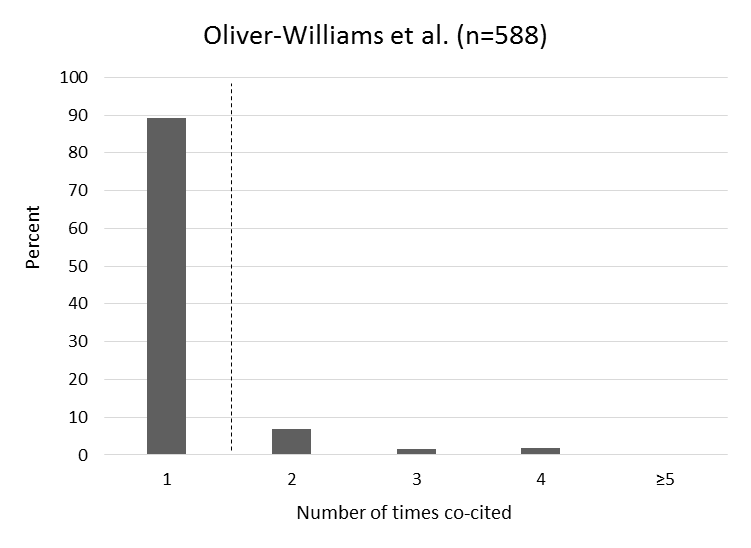

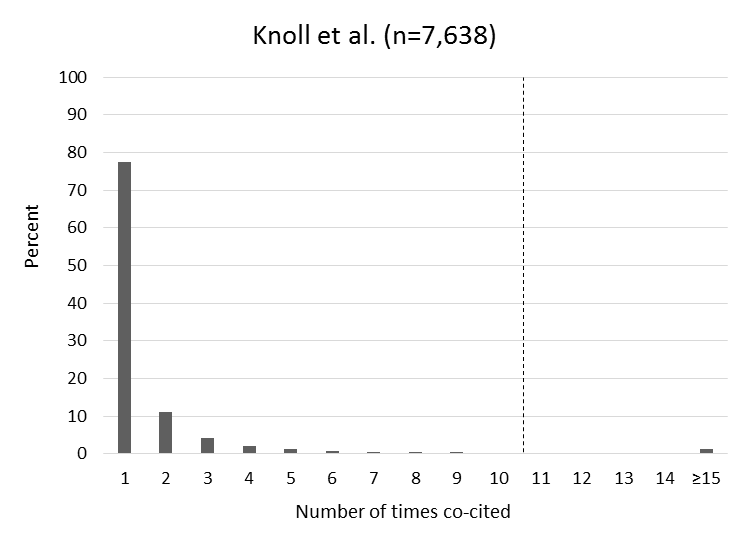

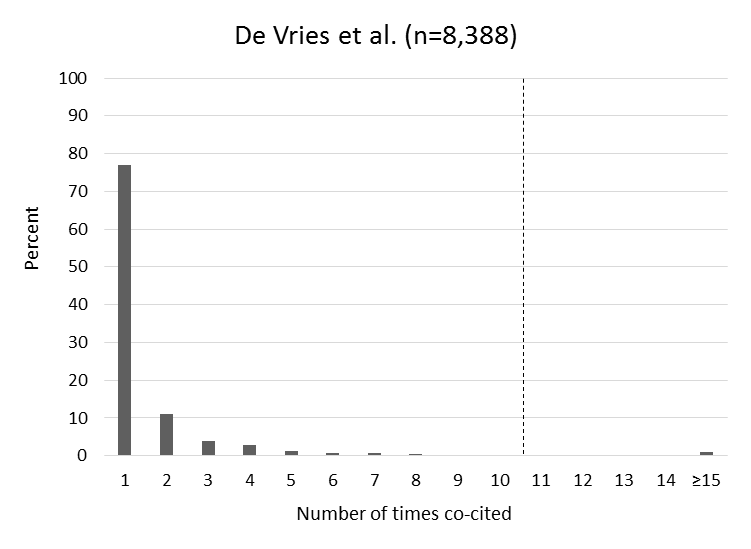

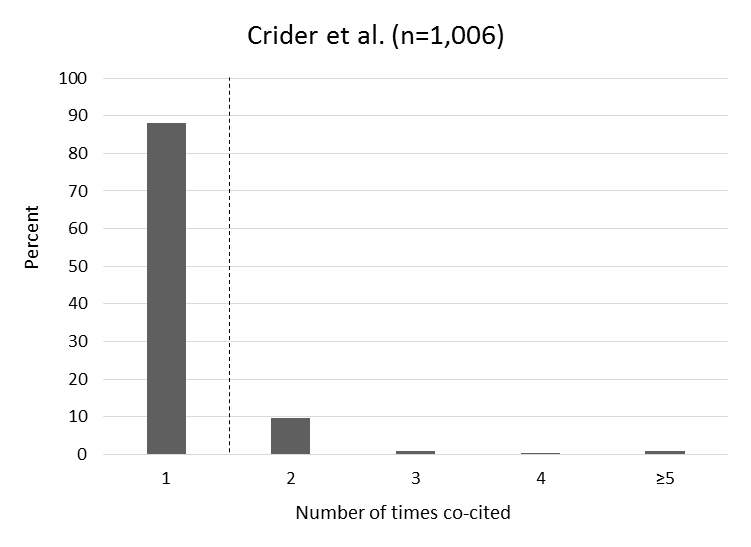

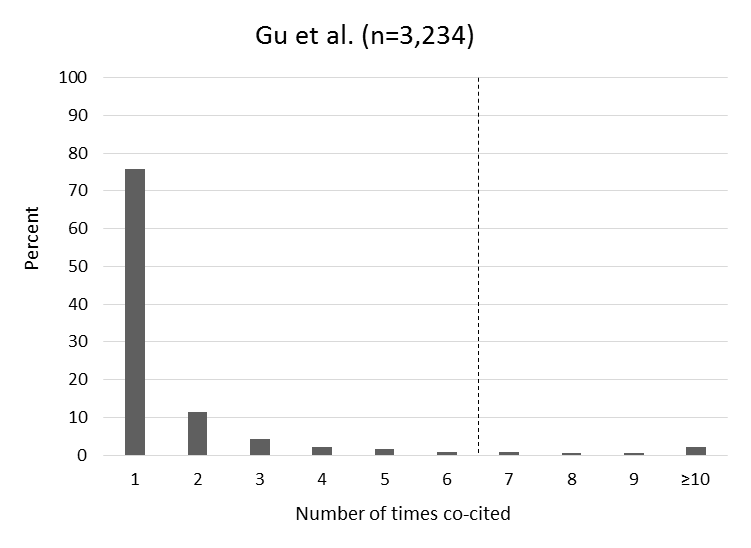

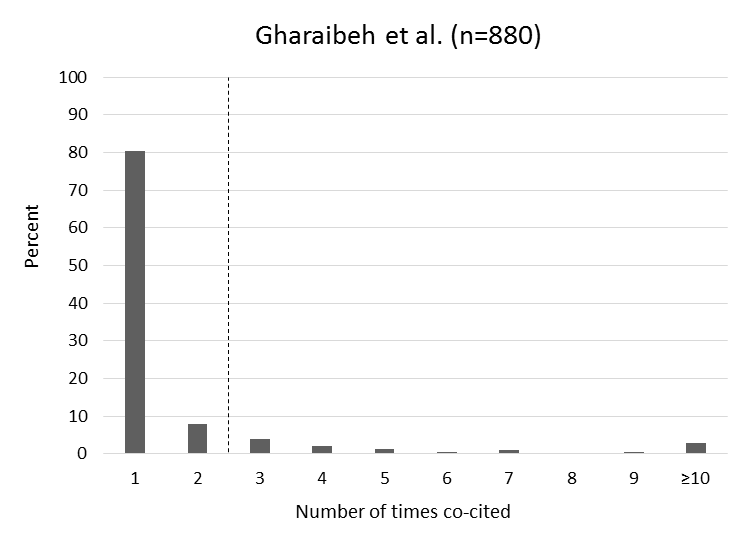

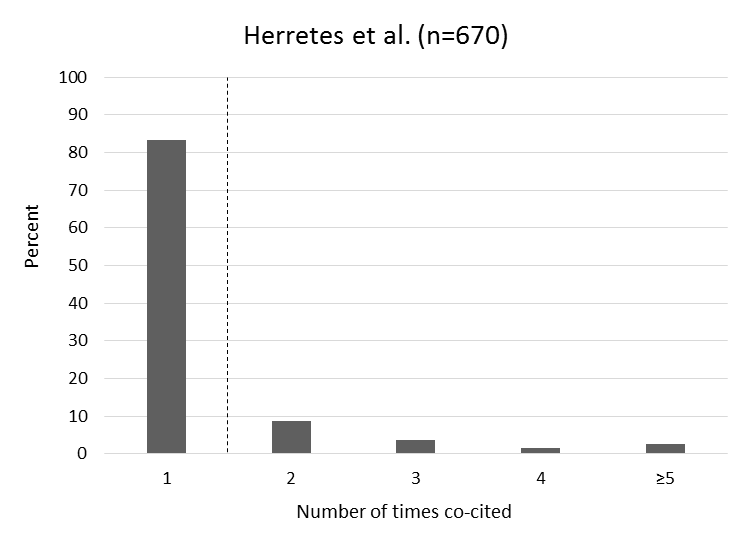

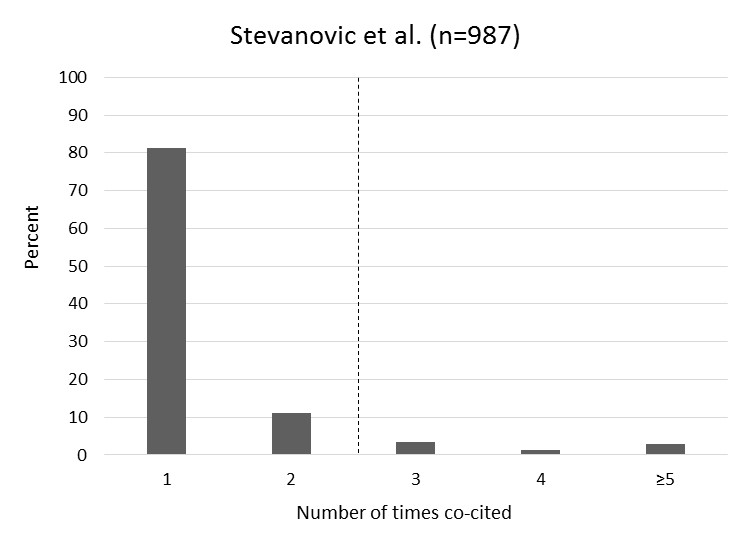

Supplement: Additional file 1: — (DOCX 975 kb) [file 12874_2015_77_MOESM1_ESM.docx]
